# Supplementary material for: Bismuth−Antimony Alloy Embedded in Carbon Matrix for Ultra-Stable Sodium Storage
Source: Materials (Basel). 2023 Mar 9;16(6):2189. doi: 10.3390/ma16062189 (PMC10051522; doi:10.3390/ma16062189)
Supplement: Supplementary file 1 [file materials-16-02189-s001.zip › materials-2256739-supplementary.pdf]

Supplementary material:

Bismuth−Antimony Alloy Embedded in Carbon Matrix for Ultra-Stable Sodium Storage

Wensheng Ma, Bin Yu, Fuquan Tan, Hui Gao * and Zhonghua Zhang *

Key Laboratory for Liquid-Solid Structural Evolution and Processing of Materials (Ministry of Education), School of Materials Science and Engineering, Shandong University, Jingshi Road 17923, Jinan 250061, China

* Correspondence: hgao199306@gmail.com (H.G.); zh_zhang@sdu.edu.cn (Z.Z.)

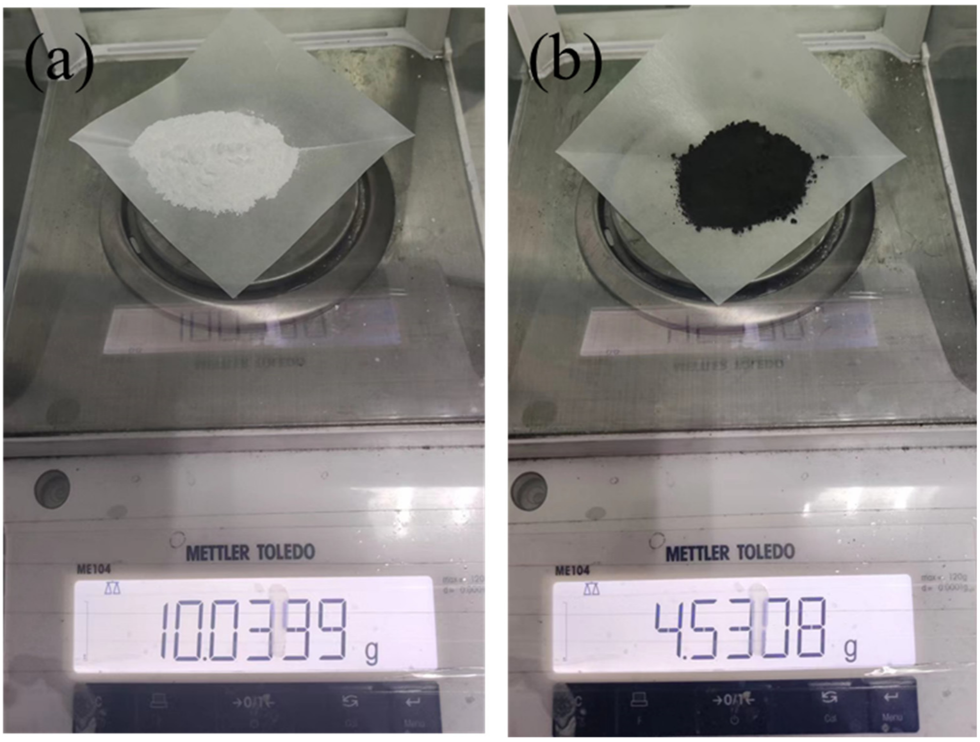

Figure S1. The photographs of (a) a mixture of bismuth citrate and antimony potassium tartrate in a molar ratio of 1:1 and (b) the obtained Bi_1Sb_1@C sample after cleaning and drying.

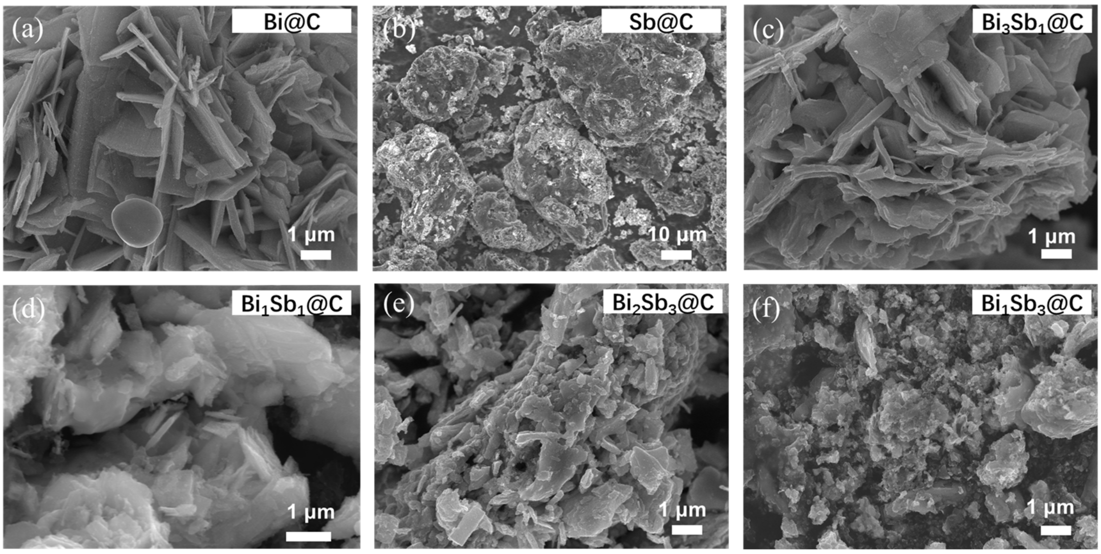

Figure S2. The SEM images at low magnifications of the (a) Bi@C, (b) Sb@C, (c) $Bi_3Sb_1@C$, (d) $Bi_1Sb_1@C$, (e) $Bi_2Sb_3@C$ and (f) $Bi_1Sb_3@C$ samples.

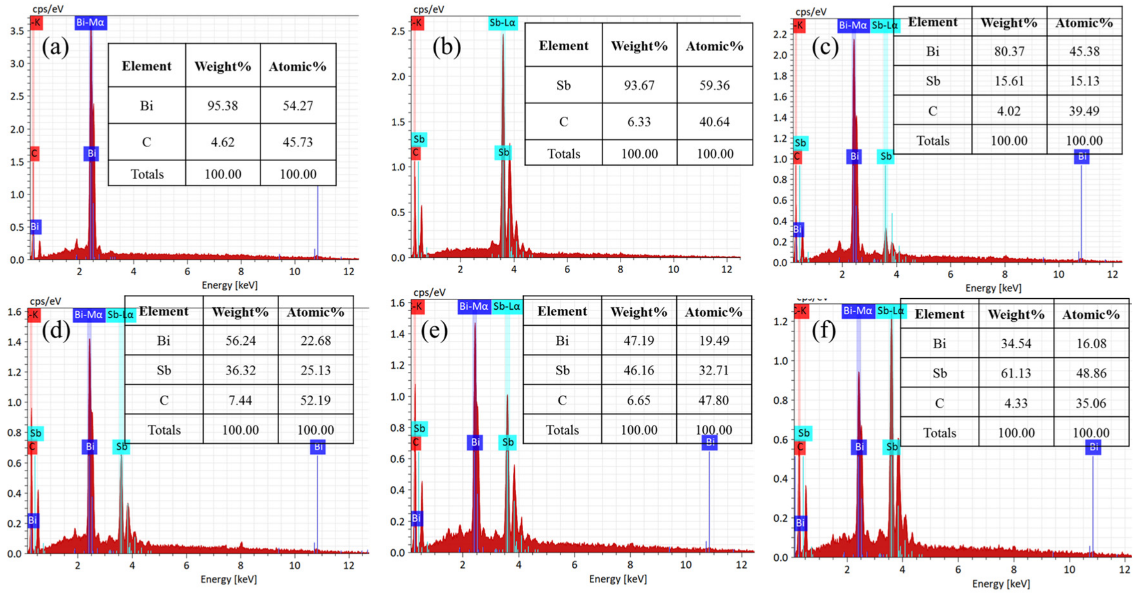

Figure S3. The corresponding EDX results of the (a)Bi@C, (b) Sb@C, (c) Bi_3Sb_1@C, (d) Bi_1Sb_1@C, (e) Bi_2Sb_3@C, and (f) Bi_1Sb_3@C samples.

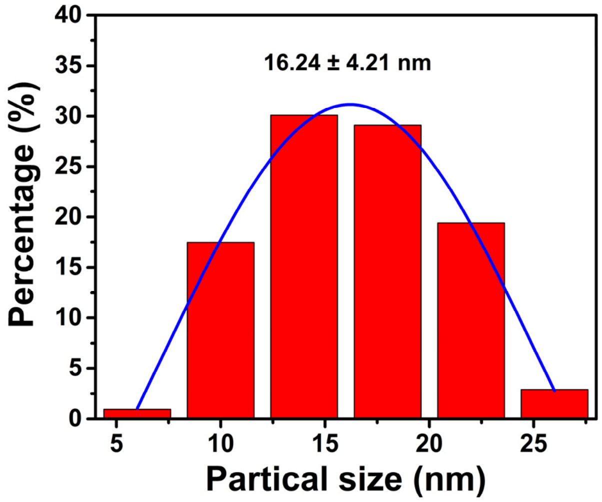

Figure S4. The size distribution pattern of the Bi_1Sb_1 alloy for the Bi_1Sb_1@C sample.

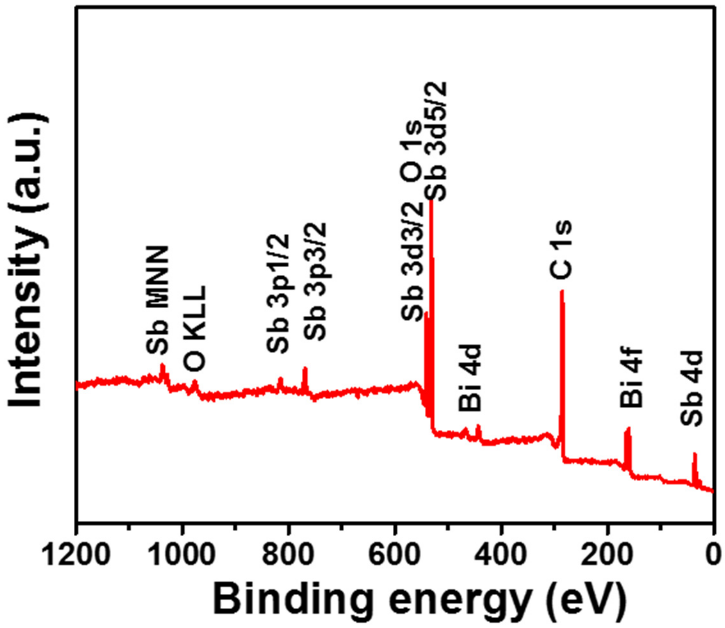

Figure S5. The low-resolution XPS spectrum of the Bi_1Sb_1@C sample.

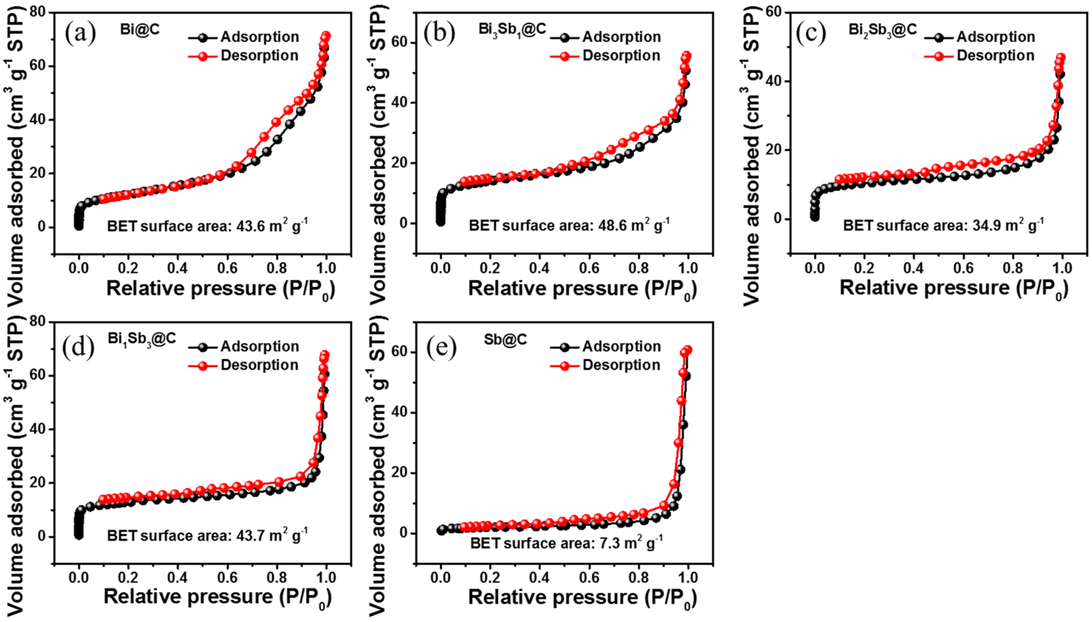

Figure S6. N_2 adsorption-desorption isotherms of the (a) Bi@C, (b) Bi_3Sb_1@C, (c) Bi_2Sb_3@C, (d) Bi_1Sb_3@C, and (e) Sb@C samples.

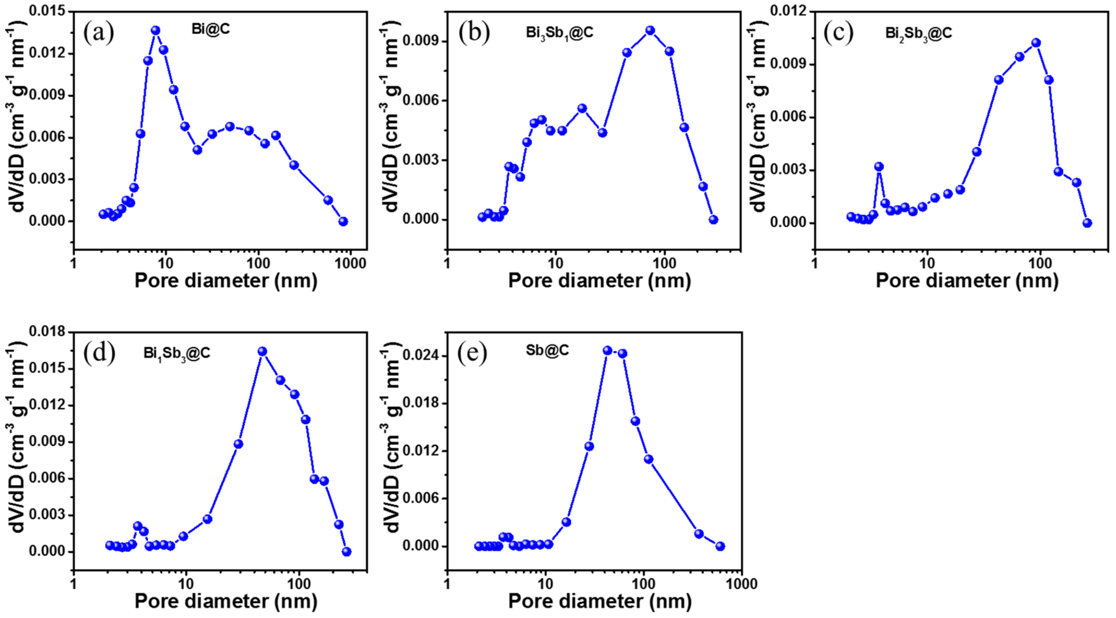

Figure S7. The pore size distribution curves of the (a) Bi@C, (b) Bi_3Sb_1@C, (c) Bi_2Sb_3@C, (d) Bi_1Sb_3@C, and (e) Sb@C samples.

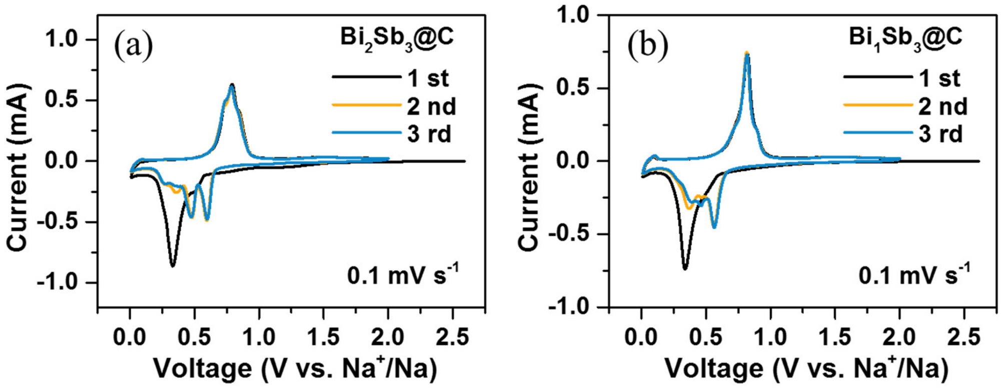

Figure S8. Initial three cycles of CV curves at a scan rate of 0.1 mV s^{-1} of the (a) Bi_2Sb_3@C and (b) Bi_1Sb_3@C samples.

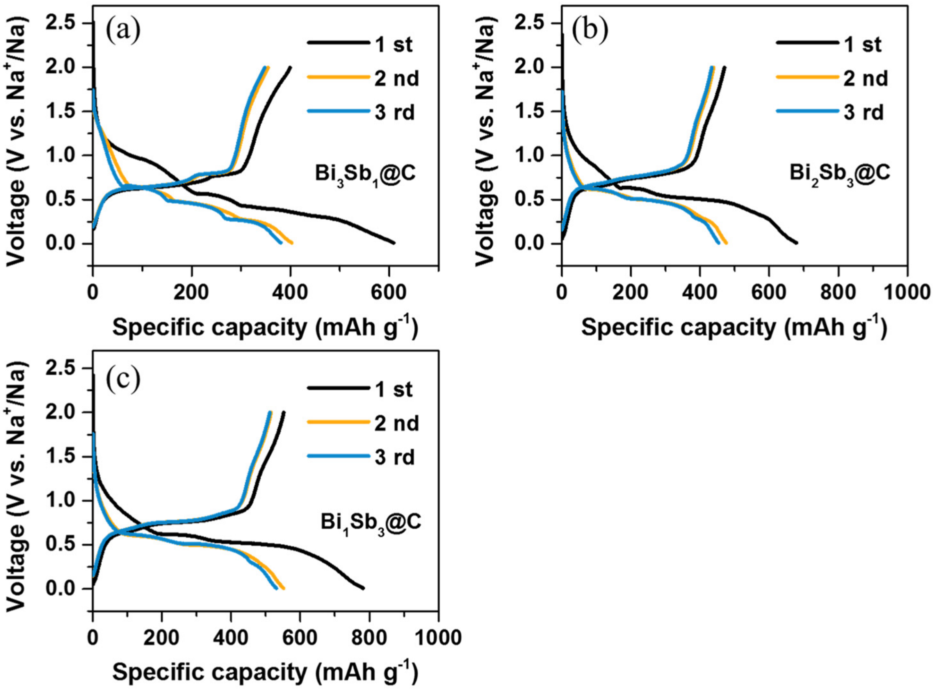

Figure S9. The discharge-charge curves of the (a) Bi_3Sb_1@C, (b) Bi_2Sb_3@C, and (c) Bi_1Sb_3@C samples during the initial three cycles.

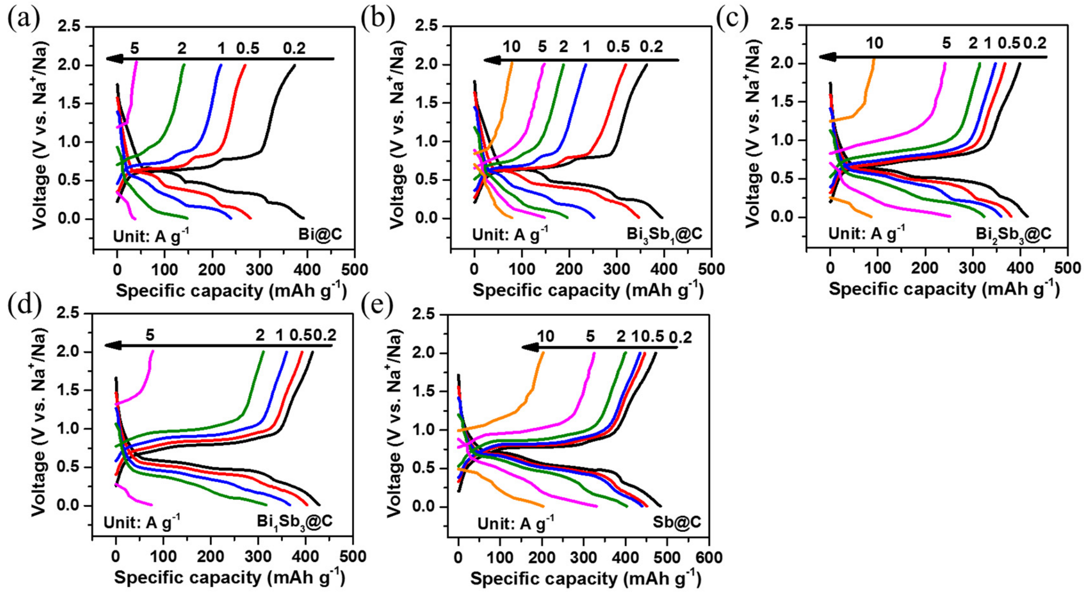

Figure S10. The discharge-charge profiles at different current densities of the (a) Bi@C, (b) Bi_3Sb_1@C, (c) Bi_2Sb_3@C, (d) Bi_1Sb_3@C, and (e) Sb@C samples.

(a) (b)

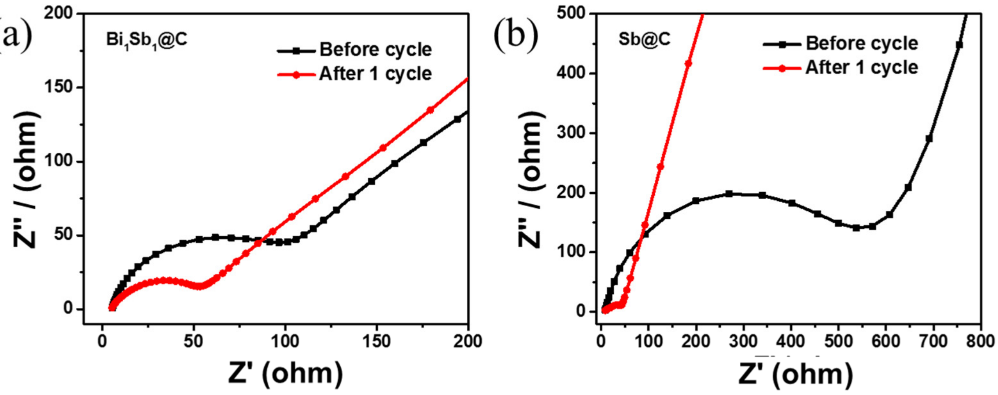

(c)

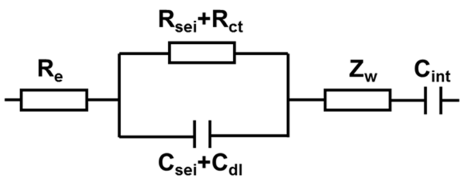

(d)

	State	R_e / ohm	R_{sei}+R_{ct} / ohm	C_{sei}+C_{dl} / F
Sb@C	Before cycle	10.5	521	1.2×10^{-6}
	After 1 cycle	11.0	52	2.5×10^{-5}
Bi_1Sb_1@C	Before cycle	9.4	98	1.9×10^{-4}
	After 1 cycle	9.4	46	1.7×10^{-4}

Figure S11. Nyquist plots of (a) Bi_1Sb_1@C and (b) Sb@C before/after the first cycle. (c, d) Fitted circuit diagram and corresponding fitting results.

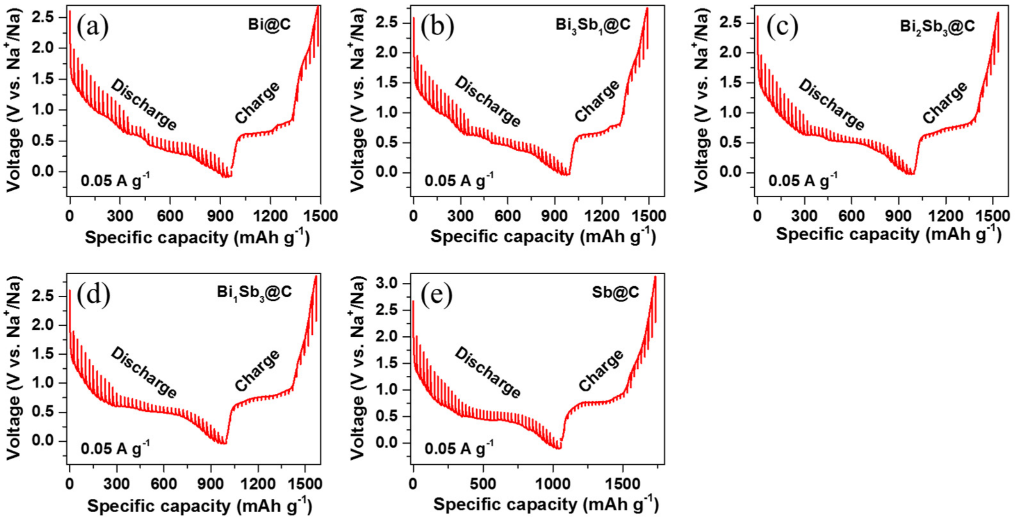

Figure S12. The GITT curves of the (a) Bi@C, (b) Bi_3Sb_1@C, (c) Bi_2Sb_3@C, (d) Bi_1Sb_3@C, and (e) Sb@C samples during the first discharge and charge processes.

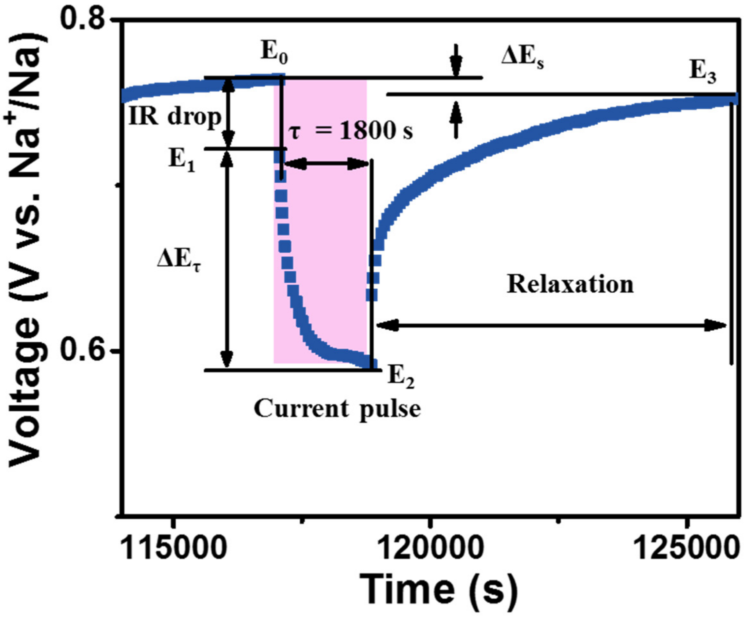

Figure S13. Schematic of one discharge pulse at ~0.75 V (vs. Na^{+}/Na) for the Bi_1Sb_1@C electrode during discharge process. ΔEs and ΔEτ represent the steady-state voltage change and transient voltage change, respectively. During a pulse discharge, the voltage rapidly drops from E_0 to E_1 due to ohmic and charge transfer resistance (IR = E_0-E_1). Then the voltage gradually decreases to E_2 due to the sodiation process of electrode. During the relaxation process, the voltage returns to the steady state (E_3). The values of ΔEs and ΔEτ are calculated by ΔEs = E_0 – E_3 and ΔEτ = E_1 – E_2, respectively.

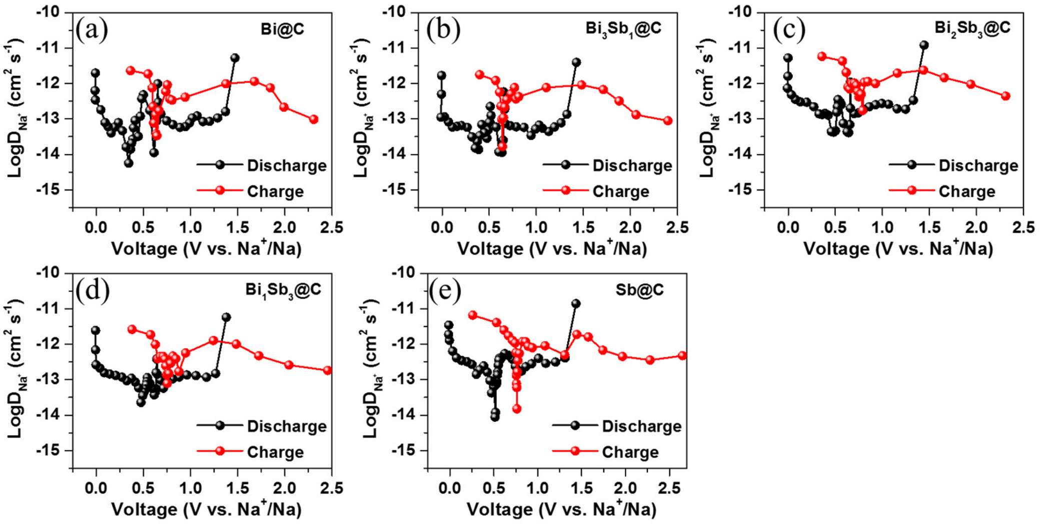

Figure S14. The diffusion rate for Na^+ of the (a) Bi@C, (b) Bi_3Sb_1@C, (c) Bi_2Sb_3@C, (d) Bi_1Sb_3@C, and (e) Sb@C samples during the first discharge and charge processes.
